# Polymorphisms of *HLA-DM* on Treatment Response to Interferon/Ribavirin in Patients with Chronic Hepatitis C Virus Type 1 Infection

**DOI:** 10.3390/ijerph13101030

**Published:** 2016-10-20

**Authors:** Hongbo Chen, Yinan Yao, Yifan Wang, Hua Zhou, Tianxiang Xu, Jing Liu, Guocheng Wang, Yongfeng Zhang, Xiang Chen, Qingwei Liu, Peng Huang, Rongbin Yu

**Affiliations:** 1 Department of Infectious Disease, the Jurong People’s Hospital, Jurong 212400, China; chb2180@126.com (H.C.); yifanwang333@163.com (Y.W.); huazhou4456@163.com (H.Z.); xutianq@163.com (T.X.); xyxJ6300@sina.com (J.L.); guochengwang4456@163.com (G.W.); yongfengzhang666@163.com (Y.Z.); xiangchen322@163.com (X.C.); lin553210@163.com (Q.L.); 2 Department of Epidemiology and Biostatistics, School of Public Health, Nanjing Medical University, Nanjing 211166, China; yinanyao1130@163.com

**Keywords:** hepatitis C, genetic polymorphism, *HLA-DM* gene, treatment outcome

## Abstract

Background: *HLA-DM* gene, which is related to antigen processing and presentation and located in the non-classical class-II region of human leukocyte antigen (HLA) region, may play a crucial role in chronic hepatitis C virus (HCV) infection treatment outcomes. The study was conducted to evaluate the role of the variant of several single nucleotide polymorphisms (SNPs) in *HLA-DM* gene in HCV treatment outcomes. Methods: We genotyped four SNPs from the candidate genes (*HLA-DMA* and *DMB*) in 336 patients who were treated with pegylated interferon-alpha and ribavirin (PEG IFN-α/RBV). Multivariate analysis of factors predicting sustained virological response (SVR) was conducted. Results: *HLA-DMA* rs1063478 and *DMB* rs23544 were independent factors of HCV treatment outcomes in Chinese Han population. Individuals who carried favorable genotypes of rs1063478TT and rs23544GG were more likely to achieve SVR {Dominant model: odds ratio (OR) = 2.05, 95% confidence interval (CI) = 1.24–3.41; OR = 2.04, 95% CI =1.23–3.35, respectively}. Rs23544, rs1063478, baseline glucose, baseline platelet and T4 level were independent predictors of SVR. The area under the receiver operating characteristic (ROC) curve (AUC) was 0.740. Conclusions: The genetic variation of rs1063478 and rs23544 are associated with the treatment outcomes in the Chinese Han population.

## 1. Introduction

Hepatitis C virus (HCV) infection is a global public health problem affecting 160 million individuals worldwide [1]. 15%–25% of HCV infections can be removed in the first six months by immune response, while 75%–85% of them become chronic and finally develop into liver cirrhosis and hepatocellular carcinoma, and part become autoimmune disorders and lymphoma [2]. Currently the approved therapy for HCV-1 is a combination of pegylated interferon (PEG-IFN) and ribavirin (RBV) for 48 and 24 weeks, respectively [3]. Success of the treatment is defined as an absence of HCV RNA 24 weeks after the cessation of therapy, and approximately 60%–70% sustained virological response (SVR) occurring in HCV-1 infection [4].

One of the possible causes of antiviral treatment failure is that viral antigen cannot be effectively recognized by T cells; therefore, the immune response can hardly be stimulated by T cells and this has protective effects [5,6]. The human leucocyte antigen (HLA) region encodes multiple genes, which participate in antigen presentation and T cell activation [7]. This region is in the short arm of chromosome 6 and those genes are divided into three categories. Genes involved in antigen processing and presentation reside on class-I and class-II genomic region, including *TAP*, *LMP*, *HLA-DM*, *HLA-DO* and *tapasin*.

Previous studies have indicated that single nucleotide polymorphisms (SNPs) of HLA classic genes were associated with the pathogenesis of many diseases, such as systemic lupus erythematosus (SLE) and human papillomavirus (HPV) related cervical cancer [8,9,10]. As is well known, *IL28B* is related to antiviral therapy. Studies about hepatitis C in the United States showed that the effectiveness of antiviral treatment for blacks and whites is related to polymorphisms of MHC class-II [11]. These studies suggested that the polymorphism of MHC molecules, especially the *HLA-DM* and *DO* genes, can be regarded as genetic markers which could assist in the diagnosis, outcome prediction and prognosis.

Antigen processing and presentation gene polymorphisms are speculated to be associated with the susceptibility and outcomes of HCV. Our earlier study discovered that *HLA-DMA* rs1063478-T mutant protects against HCV infection [12]. Thus, further research on *HLA-DM* genes should be to be conducted to reveal the possible relationships between different *HLA-DM* genotypes and treatment outcomes in the Chinese Han population with chronic hepatitis C (CHC).

## 2. Materials and Methods

### 2.1. Ethics Statement

Written informed consent was obtained from all participants in this study, the investigations were carried out following the rules of the Declaration of Helsinki, and the study protocol was checked by the Institutional Review Committee of Nanjing Medical University (2015-SRFA-105).

### 2.2. Study Subjects

A total of 336 chronic hepatitis C patients with viral genotype 1 were recruited from the Jurong People’s Hospital from January 2011 to May 2015. Eligibility criteria for therapy included: (1) age between 18 and 70 years; (2) treatment-naïve; (3) detectable HCV RNA in serum over a span of more than 6 months of treatment initiation; (4) negative for hepatitis B infection and other types of liver diseases.

All patients were treated for 48 weeks with PEG IFN-α at a dose of 180 μg subcutaneously each week plus daily 600–1000 mg of oral RBV according to the standard guidelines. Successful treatment was identified based on SVR, defined as absence of HCV RNA 24 weeks after the cessation of therapy. In this study, rapid virological response (RVR) were defined as undetectable HCVRNA at 4 weeks of therapy; Early virological response (EVR) were defined as ≥2 log reduction in HCV RNA level compared to baseline HCV RNA level or undetectable HCVRNA at 12 weeks during therapy. Complete early virological response (cEVR) indicated a positive outcome of the treatment of HCV RNA at 4 weeks, but undetectable HCVRNA after 12 weeks’ treatment.

### 2.3. Laboratory Testing

Blood samples for biochemical analysis, SNP determination, were collected before antiviral therapy. Serum viral load of all treated patients was quantified at baseline, weeks 4, 12, 24, 48, and 24 weeks after cessation of treatment by a Cobas Amplicor HCV Monitor Test (v2.0, Roche, Basel, Switzerland).

### 2.4. SNP Genotyping

Genomic DNA was extracted from peripheral blood mononuclear cells using protease K digestion and phenol-chloroform purification. The polymorphisms of rs23544, rs3135029, rs1050391 and rs1063478 were genotyped by the TaqMan allelic discrimination assay on ABI PRISM 7900HT Sequence Detection system (Applied Biosystems, San Diego, CA, USA). The primers used for genotyping are listed in Supplementary Materials Table S1. The genotyping results were determined by the allelic discrimination mode of the SDS 2.3 software package (Applied Biosystems, Foster City, CA, USA), and a 100% concordant was achieved. 

### 2.5. Statistical Analysis

Demographic characteristics of individuals were compared using two-sample *t* tests and chi-square (*χ*^2^) test as appropriate. The association of genotypes with SVR were estimated by odds ratio (OR) and 95% CI using multivariate logistic regression analysis, also adjusted for age, sex, baseline HCV-RNA level, γ-glutamyl transpeptidase (GGT), glucose, T3, T4, platelets, and α-fetal protein (AFP). Cochran-Armitage trend test was used to analyze the combined effect of two independent SNPs (rs23544-G and rs1063478-C). A stepwise regression model was then fit comprised of all variables and subsequently reduced using forwards elimination to analyze predictive factors for SVR. The predictive models of SVR were presented by receiver-operating characteristic (ROC) curve and area under the curve (AUC) represented the prediction value. In all analyses, a *p*-value of 0.05 or less was considered statistically significant. All statistical analyses were performed by Stata/SE (V.12.0 for Windows; StataCorp LP, College Station, TX, USA).

## 3. Results

### 3.1. Baseline Characteristics of the Study Population

Baseline clinical and laboratory indicators of the 336 enrolled patients are shown in Table 1. The RVR, EVR and cEVR rates were 47.3%, 81.6% and 69.5% respectively. A total of 253 patients achieved SVR (75.3%), among them, the mean age was 53.52 ± 8.57 years and 24.9% were males.

Baseline levels of viral load, GGT, glucose, T3, T4, platelets, and AFP were different between the SVR group and the non-SVR group (*p* < 0.05). Patients with high viral load, T3, T4 and abnormal GGT, glucose, AFP, platelets levels at baseline were more likely to fail in treatment.

### 3.2. Association of Polymorphisms in HLA-DM with Virological Response to Treatment

Analysis of allelic frequencies of the investigated polymorphisms showed that both cohorts were in Hardy-Weinberg equilibrium (*p* > 0.05). Each SNP was analyzed by three genetic models (Dominant, Recessive, and Additive models) to determine the effect on RVR, EVR, cEVR and SVR. All factors were adjusted with a *p-*values < 0.2 in the univariate analysis.

For patients with the rs23544GG genotype a higher SVR rate (83.3%) was observed in comparison with AG and AA genotypes (71.4% and 56.3% respectively) (Dominant model: OR = 2.04, 95% CI = 1.23–3.35) (Table 2). Similarly, significantly higher SVR rates were observed in subjects with rs1063478TT genotype with respect to those with wild-type genotype or heterozygous genotype (Dominant model: OR = 2.05, 95% CI = 1.24–3.41). Furthermore, rs23544 and rs1063478 was also found to be strongly associated with cEVR (Recessive model: OR = 2.27, 95% CI = 1.02–5.04; Dominant model: OR = 1.70, 95% CI = 1.02–2.82, respectively) (Supplementary Materials Table S2). We also found that patients with rs1063478 TT genotype may be prone to achieve a higher RVR rate (Recessive model: OR = 2.65, 95% CI = 1.16–6.06) (Supplementary Materials Table S2).

Subsequently, we evaluated combined effects by adding up the number of variant alleles of the two SNPs. The results showed that SVR rate increased when patients carrying more favorable rs23544 G and rs1063478 T alleles (Table 3).

### 3.3. Predictive Factors for SVR

**Figure 1:**
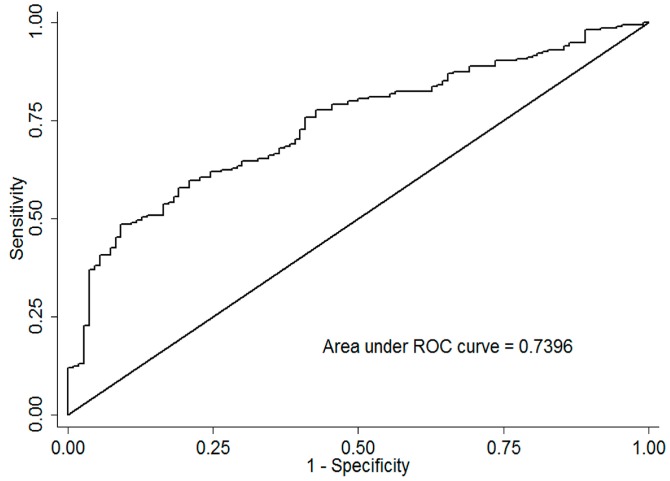
Predictors of hepatitis C virus treatment response.

As shown in Table 4, a stepwise regression model comprised of all variables was established. Rs23544, rs1063478, baseline glucose, baseline platelet and T4 level were independent predictors of SVR. When including one SNP of rs1063478, a relatively small area was obtained (AUC = 0.587). A bigger AUC of 0.619 was yield when combined with one SNP of rs23544, which suggested that the prediction value of rs23544 was stronger than rs1063478. Adding up the five factors of rs23544, rs1063478, baseline glucose, platelet and T4 level, the prediction value increased to 0.74 (Figure 1).

## 4. Discussion

We selected and analyzed 4SNPs in *HLA-DM* genes, which were related to antigen processing and presentation. The results showed that *HLA-DMB* rs23544 and *HLA-DMA* rs1063478 polymorphism were associated with HCV treatment outcomes. Rs1063478-T and rs23544-G mutants were biomarkers of SVR. *HLA-DMA* rs1063478 (C > T) makes a missense mutation and *HLA-DMB* rs23544 (A > G) located in intron region. The two SNPs are both in *HLA-DM* gene and the rs1063478-Tmutation has the potential to alter the function of the encoding protein and influence the role of *DM* gene in antigen processing and presentation. Thus, the mutations may further affect the remove of the virus and influence the achievement of SVR. The combined analysis of the two SNPs indicated that the SVR rate increased when patients carried more favorable rs23544 G and rs1063478 T alleles. The range of confidence interval carrying more than three favorable alleles was relatively wide due to the limited sample size of each group. However, the predictive value of rs1063487-T (AUC = 0.587) was smaller than rs23544-G (AUC = 0.619). According to the stepwise regression analysis, rs23544, rs1063478, baseline glucose, baseline platelet and T4 level were independent predictors of SVR. When including these five factors, the prediction value AUC was 0.74. This predictive model was similar to several previous studies and it could help with the judgment of prognosis and adjustment for therapy regimen [13,14,15].

Sanchez et al. discovered that variation in the *HLA-DM* gene was relevant in producing pathogenicautoantibodies-antiphospholipid antibodies (aPL) in 2004 [8]. In 2013 Zhang et al. reported the association between *HLA-DM* gene and childhood systemic lupus erythematosus [16]. Aissani et al. reported that *HLA-DMB* can be a candidate susceptibility gene for HIV-related Kaposi’s sarcoma in 2014 [17]. All these studies proved that the polymorphisms of *HLA-DM* gene are related to diseases of the immune system. Our previous study on HCV susceptibility found that rs1063478 was significant [12]. Thus, rs1063478 played an important role both in HCV susceptibility and treatment outcomes; also, *HLA-DM* gene was associated with HCV treatment outcomes.

Our treatment cohort had good representativeness because all the patients enrolled in this study were only infected with HCV and from the same district. Besides, a total of 336 patients were included in this study, so the sample size was relatively large, but it has to be pointed out that there are several potential limitations in our study. First, we only genotyped four SNPs in *HLA-DM* gene and only two SNPs were determined. Second, the relationship between the polymorphism of *HLA-DM* gene and treatment outcomes has so far not been explained. It might be associated with different ethnicities. To solve this problem, further studies in racially diverse populations are needed. Last but not least, in the study, only the response to IFN-free regimen has been predicted, but nowadays triple direct antiviral (DAA) therapy is the trend for HCV infection treatment, and an IFN-free regimen, which is at the expense of adverse effects and high costs, has already been widely used in developed countries. In some developing countries, like China, IFN-based therapy is still the first-line therapy for HCV-1 patients [18]. As a result, researches on IFN-based therapy still have certain significance in Chinese population, and DAA could be a direction of future research.

## 5. Conclusions

This study showed that the genetic variants of *HLA-DM* gene may have an important role in treatment response in the Chinese population. Rs23544, rs1063478, baseline glucose, baseline platelet and T4 level were independent predictors of SVR. Rs1063478 located in the missense mutation region and may play an important role in changing the function. Further research should be conducted to elucidate the mechanism of the SNPs.

## Figures and Tables

**Table 1:** Baseline characteristics of chronic hepatitis C patients treated with IFN/RBV.

Variables	N-SVR	SVR	*p*-Value
	(*n* = 83)	(*n* = 253)	
Mean age, years	53.60 ± 8.05	53.52 ± 8.57	0.917
Age ≥ 50	77 (69.4)	151 (67.1)	0.677
Male (%)	27 (24.3)	56 (24.9)	0.910
AST ≥ 40 U/L (%)	61 (54.9)	109 (48.4)	0.262
ALT ≥ 40 U/L (%)	67 (63.4)	125 (55.6)	0.403
GGT ≥ 50 (U/L)	50 (45.1)	71 (31.6)	0.015
GLU > 6 (mmol/L)	49 (44.1)	58 (25.8)	0.001
AFP > 7.02 (ng/mL)	36 (32.4)	45 (20.0)	0.012
T3 (nmol/L)	1.55 ± 0.44	1.41 ± 0.45	0.008
T4 (nmol/L)	130.94 ± 33.45	121.55 ± 30.48	0.011
Anti-TPO ≥ 35 I/mL	16 (14.5)	33 (15.0)	0.913
baseline HCV-RNA	6.21 ± 0.74	6.00 ± 0.85	0.032
TP (g/L)	79.79 ± 5.84	78.84 ± 6.80	0.061
ALB (g/L)	43.34 ± 4.25	43.84 ± 4.29	0.316
Platelets (10^9^/L)	133.47 ± 68.85	147.48 ± 60.63	0.058
Abnormal	47 (42.3)	58 (25.9)	0.002
Normal	64 (57.7)	166 (74.1)	
WBC (10^9^/L)	4.79 ± 1.79	4.96 ± 1.72	0.394
Abnormal	43 (39.1)	72 (32.0)	0.199
Normal	67 (60.9)	153 (68.0)	
Hemoglobin (g/L)	133.52 ± 17.86	132.94 ± 17.55	0.782
Abnormal	31 (27.9)	56 (24.9)	0.550
Normal	80 (72.1)	169 (75.1)	

Abbreviation: IFN, interferon; RBV, ribavirin; N-SVR, non-sustained virological response; SVR, sustained virological response; AST, aspartate transaminase; ALT, alanine aminotransferase; GGT, gamma-glutamyl transpeptidase; GLU, glucose; AFP, alpha fetal protein; TP, total protein; ALB, albumin; WBC, white blood cell.

**Table 2:** Association of SNPs in *HLA-DM* with SVR.

Genotype	N-SVR	SVR	SVR Rate (%)	OR (95% CI)	*p*-Value
rs23544					
AA	62 (55.9)	80 (35.6)	56.3	1.00	-
AG	40 (36.0)	100 (44.4)	71.4	1.74 (1.02–2.98)	0.044
GG	9 (8.1)	45 (20.0)	83.3	3.22 (1.41–7.35)	0.006
Dominant				2.04 (1.23–3.35)	0.005
Recessive				2.52 (1.14–5.58)	0.022
Additive				1.78 (1.23–2.56)	0.002
rs3135029					
AA	71 (64.0)	155 (68.9)	68.6	1.00	-
AC	32 (28.8)	63 (28.0)	66.3	1.04 (0.59–1.83)	0.879
CC	8 (7.2)	7 (3.1)	46.7	0.37 (0.11–1.19)	0.097
Dominant				0.89 (0.53–1.51)	0.686
Recessive				0.36 (0.11–1.17)	0.090
Additive				0.81 (0.52–1.24)	0.337
rs1050391					
CC	72 (64.9)	156 (69.3)	68.4	1.00	-
CT	33 (29.7)	62 (27.6)	65.3	0.96 (0.55–1.69)	0.906
TT	6 (5.4)	7 (3.1)	53.8	0.40 (0.12–1.37)	0.148
Dominant				0.86 (0.51–1.46)	0.577
Recessive				0.41 (0.12–1.37)	0.149
Additive				0.80 (0.52–1.24)	0.331
rs1063478					
CC	59 (53.2)	86 (38.2)	59.3	1.00	-
CT	46 (41.4)	111 (49.3)	70.7	1.83 (1.08–3.08)	0.022
TT	6 (5.4)	28 (12.5)	82.3	4.40 (1.48–13.03)	0.007
Dominant				2.05 (1.24–3.41)	0.005
Recessive				3.15 (1.11–8.96)	0.031
Additive				1.96 (1.29–2.96)	0.002

Logistic regression analyses adjusted for age, gender, gamma-glutamyl transpeptidase, glucose, α-fetal protein, platelets, baseline RNA, T3, T4; Abbreviation: SNP, single nucleotide polymorphism; SVR, sustained virological response; N-SVR, non-sustained virological response; OR, odds ratio; CI, confidence interval; -, reference. Dominant model stands for (homozygous type + hybrid type) vs. wild type; recessive model stands for homozygous type vs. (hybrid type + wild type) and additive model stands for hybrid type vs. homozygous type vs. wild type.

**Table 3:** Combined effects of rs23544 and rs1063478 with SVR.

Variables	N-SVR	SVR	SVR Rate (%)	OR (95% CI)	*p*-Value
0	28 (25.2)	22 (9.8)	44.0	1.00	-
1	51 (46.0)	94 (41.8)	64.8	2.27 (1.10–4.63)	0.025
2	31 (27.9)	74 (32.9)	70.5	2.91 (1.37–6.17)	0.005
3–4	1 (0.9)	35 (15.5)	97.2	45.12 (5.49–370.75)	<0.001
Trend					^a^ *p* < 0.001

Variables are numbers of combined favorable mutants (rs23544-G and rs1063478-C); Logistic regression analyses adjusted for age, gender, gamma-glutamyl transpeptidase, glucose, α-fetal protein, platelets, baseline RNA, T3, T4; Abbreviation: SVR, sustained virological response; N-SVR, non-sustained virological response; OR, odds ratio; CI, confidence interval; ^a^ *p*-value was analyzed by Cochran-Armitage trend test.

**Table 4:** Multivariate Stepwise regression analysis for independent factors of SVR.

Variables	Coef.	SE	OR (95% CI)	*p*-Value
rs23544	0.68	0.37	1.98 (1.36–2.89)	<0.001
rs1063478	0.79	0.47	2.22 (1.45–3.38)	<0.001
GLU	−0.77	0.12	0.46 (0.27–0.78)	0.004
Platelets (10^9^/L)	0.70	0.52	2.02 (1.21–3.34)	0.007
T4	−0.008	0.004	0.99 (0.98–1.00)	0.046

Abbreviation: SVR, sustained virological response; Coef., coefficient of variation; SE, standard error; OR, odds ratio; CI, confidence interval; GLU, glucose.

## Supplementary Materials

The following are available online at www.mdpi.com/1660-4601/13/10/1030/s1, Table S1: Information of primers and probes for TaqMan allelic discrimination; Table S2: Association of SNPs in *HLA-DM* with EVR and cEVR.
