# Supplementary material for: Polymorphisms of HLA-DM on Treatment Response to Interferon/Ribavirin in Patients with Chronic Hepatitis C Virus Type 1 Infection
Source: Int J Environ Res Public Health. 2016 Oct 20;13(10):1030. doi: 10.3390/ijerph13101030 (PMC5086769; doi:10.3390/ijerph13101030)
Supplement: Supplementary file 1 [file ijerph-13-01030-s001.pdf]

Supplementary Materials: Polymorphisms of *HLA-DM* on Treatment Response to Interferon/Ribavirin in Patients with Chronic Hepatitis C Virus Type 1 Infection

Hongbo Chen, Yinan Yao, Yifan Wang, Hua Zhou, Tianxiang Xu, Jing Liu, Guocheng Wang, Yongfeng Zhang, Xiang Chen, Qingwei Liu, Peng Huang and Rongbin Yu

Table S1. Primers and probes for TaqMan allelic discrimination.

Polymorphism		Sequence(5′-3′)
rs23544	Primer	F: CCACTGTATTAGAGAGGACCTGGAA
		R: ACCACCCACTCCCAAGAAAAT
	Probe	FAM-AGATGGATTCCCC-MGB
		HEX-AGATGGGTTCCCC-MGB
rs3135029	Primer	F: TCGCTTGGGTGTTCCTGTATAA
		R: CAGGGCCCACGTGATCTG
	Probe	FAM-TAAGCTACAAATTCTGGCA-MGB
		HEX-ATAAGCTACAAATTCGGGCAA-MGB
rs1050391	Primer	F: AGGTCTTCTTCCAGGCAAGGA
		R: GAGCTATAGACAGGAAGTGCTGAA
	Probe	FAM-CTAGACGTAGAAGCAG-MGB
		HEX-AGCTAGACATAGAAGCAGA-MGB
rs1063478	Primer	F: CCACCCATGCTGACAGTGA
		R: AGTCCATCGACAGCTGAGACA
	Probe	FAM-ATCATTCCGTCCCTGTG-MGB
		HEX-CATCATTCCATCCCTGT-MGB

Table S2. Association of SNPsin *HLA-DM* with EVR and cEVR.

Genotype	N-RVR	RVR	OR (95% CI)	*p*-Value	N-cEVR	cEVR	OR (95% CI)	*p*-Value
	n = 166	*n* = 149			*n* = 98	*n* = 223		
**rs23544**								
AA	73 (44.0)	61 (40.9)	1.00	-	43 (43.9)	92 (41.2)	1.00	-
AG	71 (42.8)	61 (40.9)	0.99 (0.59–1.67)	0.976	46 (46.9)	88 (39.5)	0.86 (0.49–1.48)	0.584
GG	22 (13.2)	27 (18.1)	1.30 (0.65–2.63)	0.455	9 (9.2)	43 (19.3)	2.11 (0.91–4.89)	0.081
Dominant			1.07 (0.66–1.73)	0.771			1.08 (0.65–1.79)	0.777
Recessive			1.31 (0.68–02.52)	0.420			2.27 (1.02–5.04)	0.044
Additive			1.11 (0.79–1.54)	0.544			1.25 (0.87–1.79)	0.221
**rs3135029**								
AA	116 (69.9)	96 (64.4)	1.00	-	69 (70.4)	145 (65.0)	1.00	-
AC	45 (27.1)	45 (30.2)	1.27 (0.74–2.18)	0.393	25 (25.5)	67 (30.0)	1.59 (0.89–2.87)	0.118
CC	5 (3.0)	8 (5.4)	1.83 (0.54–6.22)	0.328	4 (4.1)	11 (5.0)	1.45 (0.41–5.08)	0.566
Dominant			1.33 (0.79–2.23)	0.277			1.57 (0.90–2.75)	0.109
Recessive			1.72 (0.51–5.74)	0.379			1.26 (0.36–4.35)	0.713
Additive			1.30 (0.85–2.00)	0.224			1.41 (0.88–2.25)	0.147
**rs1050391**								
CC	116 (69.9)	97 (65.1)	1.00	-	68 (69.4)	148 (66.4)	1.00	-
CT	46 (27.7)	45 (30.2)	1.24 (0.72–2.14)	0.426	27 (27.5)	65 (29.1)	1.32 (0.74–2.34)	0.342
TT	4 (2.4)	7 (4.7)	1.73 (0.46–6.54)	0.416	3 (3.1)	10 (4.5)	1.53 (0.37–6.24)	0.553
Dominant			1.29 (0.77–2.17)	0.327			1.34 (0.78–2.33)	0.291
Recessive			1.63 (0.44–6.10)	0.463			1.42 (0.35–5.73)	0.092
Additive			1.27 (0.82–1.97)	0.282			1.29 (0.80–2.06)	0.053
**rs1063478**								
CC	70 (42.2)	65 (43.6)	1.00	-	52 (53.1)	88 (39.5)	1.00	-
CT	86 (51.8)	60 (40.3)	0.78 (0.47–1.29)	0.339	41 (41.8)	108 (48.4)	1.57 (0.93–2.64)	0.092
TT	10 (6.0)	24 (16.1)	2.31 (0.96–5.53)	0.059	5 (5.1)	27 (12.1)	2.85 (0.99–8.23)	0.053

Dominant	0.94 (0.58–1.52)	0.793	1.70 (1.02–2.82)	0.040
Recessive	2.65 (1.16–6.06)	0.021	2.25 (0.80–6.30)	0.122
Additive	1.19 (0.82–1.72)	0.357	1.63 (1.07–2.46)	0.021

Abbreviation: SNP, single nucleotide polymorphism; RVR, rapid virological response; N-RVR, non rapidvirological response. OR, odds ratio; CI, confidence interval; EVR, early virological response; cEVR, complete early virological response; N-cEVR, non-complete early virological response; -, reference; Logistic regression analyses adjusted for age, gender, gamma-glutamyltranspeptidase, glucose, α-fetal protein, platelets, baseline RNA, T3, T4. Dominant model stands for (homozygous type + hybrid type) vs. wild type; recessive model stands for homozygous type vs. (hybrid type + wild type) and additive model stands for hybrid type vs. homozygous type vs. wild type.

© 2016 by the authors; licensee MDPI, Basel, Switzerland. This article is an open access article distributed under the terms and conditions of the Creative Commons by Attribution (CC-BY) license (http://creativecommons.org/licenses/by/4.0/).
